# 294. The Impact of Mucositis on the Oral Microbiome and Clinical Outcomes in Allogenic Hematopoietic Stem Cell Transplantation

**DOI:** 10.1093/ofid/ofad500.366

**Published:** 2023-11-27

**Authors:** Julia A Messina, Danting Jiang, Bharathi Selvan, Lauren Hill, Amy Bush, John Bokman, Tessa Andermann, Ernya Johnson, Nelson Chao, Neeraj Surana, Anthony Sung

**Affiliations:** Duke University, Apex, North Carolina; Duke University, Apex, North Carolina; Duke University School of Medicine, Durham, North Carolina; Duke University, Apex, North Carolina; Duke University, Apex, North Carolina; Duke Unviersity, Durham, North Carolina; University of North Carolina at Chapel Hill, Chapel Hill, North Carolina; Duke University, Apex, North Carolina; Duke University, Apex, North Carolina; Duke University, Apex, North Carolina; Duke University, Apex, North Carolina

## Abstract

**Background:**

Mucositis is a common complication of allogeneic hematopoietic stem cell transplantation (allo HCT) that can lead to debilitating pain, malnutrition, and bloodstream infections (BSI). We sought to characterize the oral microbiome of allo HCT recipients and correlate the oral microbiome with the development of mucositis and clinical outcomes.

**Methods:**

We collected weekly oral swabs and clinical data from patients who underwent allo HCT from 2017-21. Swabs underwent 16S rRNA sequencing, amplifying the V4 region. Patients with paired pre-HCT (prior to conditioning) and day +30 samples were included in the microbiome analysis.

**Results:**

91 patients were enrolled with 178 swabs sequenced. Mucositis occurred in 66/91 patients (73%) at a median 6 days post-HCT (Table 1). Patients with mucositis were significantly more likely to receive myeloablative conditioning, methotrexate/calcineurin inhibitor for graft-versus host disease (GVHD) prophylaxis, and total parenteral nutrition. There was not a significant difference in BSI rates at day +30 between patients with mucositis and those without (20% vs. 17%; p=0.69), but there was a trend toward more acute GVHD by day +30 in the mucositis group (27% vs. 12%; p=0.12). Additionally, patients with mucositis had lower rates of relapse (18% vs. 36%; p=0.07) and all-cause mortality at 1-year post-HCT (26% vs. 36%; p=0.33) although these comparisons were not statistically significant.

39 patients had paired 16S pre- and Day +30 data (28 with mucositis, 11 without). While there was no change in α-diversity in patients who did not develop mucositis, the development of mucositis was associated with a decrease in α-diversity (Figure 1). Although β-diversity of the pre-HCT samples was not different between the two groups, patients with mucositis had a lower pre-HCT abundance of *Veillonella parvula*.

**Figure:**
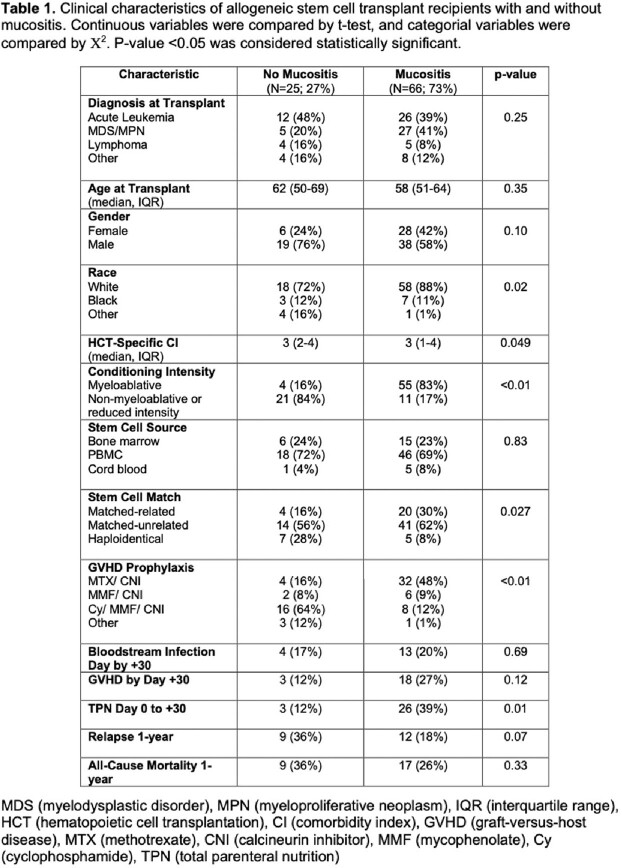

**Figure:**
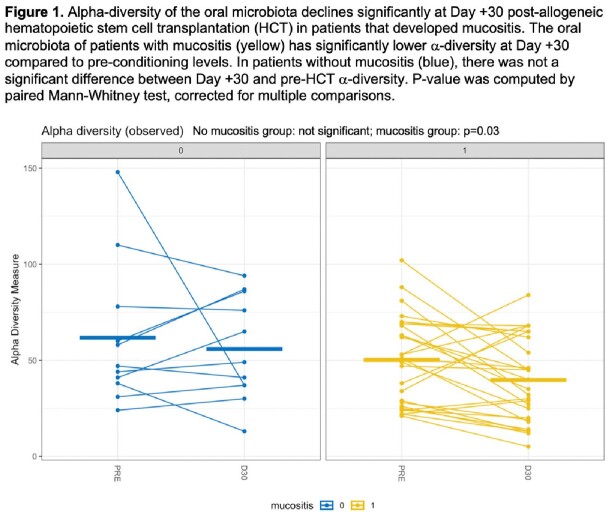

**Conclusion:**

Post-HCT mucositis led to a reduction in day +30 microbiota diversity, with *Veillonella parvula* potentially being a biomarker for the development of mucositis. Mucositis was not associated with more BSIs or higher 1-year mortality. Future study includes analysis of the gut microbiome in this same cohort to identify microbial targets that attenuate mucositis without impacting the therapeutic efficacy of HCT.

**Disclosures:**

**Neeraj Surana, MD, PhD**, Pfizer: Stocks/Bonds **Anthony Sung, MD**, clasado: Grant/Research Support|dsm: Grant/Research Support|merck: Grant/Research Support

